# The relationship between vitamin D status and islet function in patients with type 2 diabetes mellitus

**DOI:** 10.1186/s12902-021-00862-y

**Published:** 2021-10-18

**Authors:** Hang Zhao, Chong Zheng, Miaomiao Zhang, Shuchun Chen

**Affiliations:** 1 Department of Endocrinology, Hebei General Hospital, 348, Heping West Road, Shijiazhuang, 050051 Hebei China; 2 Pediatric Orthopaedics, Shijiazhuang the Third Hospital, 15, Sports South Street, Shijiazhuang, 050011 Hebei China; 3 Graduate School of Hebei Medical University, 361, Zhongshan East Road, Shijiazhuang, 050017 Hebei China

**Keywords:** Vitamin D, Islet function, Homeostasis model assessment-β, Type 2 diabetes

## Abstract

**Background:**

The aim of the study was to explore the relationship between vitamin D status and islet function in patients with type 2 diabetes mellitus.

**Methods:**

The participants were recruited from Hebei General Hospital. Basic characteristics and blood indicators were collected after fasting overnight. The data were analyzed statistically using SPSS 22.0. Analysis of variance, a nonparametric test, or a trend Chi-square test was used for the comparisons. The association between 25-hydroxy vitamin D and modified homeostasis model assessment-β was assessed using multivariate ordinal logistic regression.

**Results:**

One hundred seventy-four patients aged 26 to 79 years with type 2 diabetes mellitus were included in this study. Patients with vitamin D deficiency had a lower modified homeostasis model assessment-β level compared with those without vitamin D deficiency. There were differences in body mass index, diabetes course, glycosylated hemoglobin, fasting blood glucose, fasting blood C-peptide, triglyceride, and 25-hydroxy vitamin D among different modified homeostasis model assessment-β groups based upon the tertiles. 25-hydroxy vitamin D, as continuous or categorical variables, was positively related to modified homeostasis model assessment-β whether or not cofounding factors were adjusted.

**Conclusion:**

There is an association between increased 25-hydroxy vitamin D levels and improvement in modified homeostasis model assessment-β function in patients with type 2 diabetes mellitus.

**Trial registration:**

Cross-sectional trails ChiCTR2000029391, Registration Date: 29/01/2020.

**Supplementary Information:**

The online version contains supplementary material available at 10.1186/s12902-021-00862-y.

## Background

Widespread type 2 diabetes mellitus (T2DM) has caused a burden worldwide. Evaluation of islet function is important for patients with T2DM because it is usually used to assess the progress of T2DM and to guide drug usage. Traditionally, homeostasis model assessment (HOMA)-β has been used to evaluate islet function using fasting blood glucose (FBG) and insulin levels [1], but this is only used in patients who have not received insulin therapy; therefore, its application is limited for patients who have received injected insulin. Li [2] found that a modified HOMA-β that replaces insulin with fasting C-peptide (FCP) could be used in all patients whether or not they have taken insulin.

The relationship between vitamin D and numerous diseases including metabolic abnormalities such as insulin resistance (IR) and T2DM has been identified over time [3]. It is generally recognized that 25-hydroxy vitamin D (25OHD) represents the patient’s vitamin D status. Vitamin D deficiency has been defined as 25OHD < 20 ng/mL and vitamin D insufficiency has been defined as 25OHD < 30 ng/mL [4]. Vitamin D insufficiency or deficiency was strongly related with accelerated development of IR [5]. A low 25OHD level was related to an increased risk of T2DM and poor glucose control. A meta-analysis of randomized controlled trials (RCTs) showed that vitamin D supplementation decreased glycosylated hemoglobin (HbA1c) and FBG levels in T2DM patients with vitamin D deficiency or without obesity However, for patients with vitamin D insufficiency or sufficiency, vitamin D supplementation did not change the status of glycemic control [6]. Furthermore, low 25OHD level increased the risk or the severity of complications, such as diabetic peripheral neuropathy [7] and diabetic retinopathy [8].

Previous studies explored the relationship between vitamin D status and islet function using traditional HOMA-β. The aim of the present study was to explore the association between 25OHD and the modified HOMA-β in patients with T2DM.

## Materials and methods

### Study design

This was a cross-sectional study. This study was registered at Clinical Trial (Registration Number: ChiCTR2000029391, Registration Date: 29/01/2020) and approved by the Hebei General Hospital Ethics Committee. It was performed in accordance with the Declaration of Helsinki.

### Study population

The population was from the Department of Endocrinology of Hebei General Hospital, China. All participants were hospitalized between June 2018 and December 2019. We included adult patients (≥18 years) with T2DM. A diagnosis of diabetes mellitus (DM) was made on the basis of the World Health Organization definition, as follows: FPG level ≥ 7.0 mmol/L or 2-h post-load value ≥11.1 mmol/L. Patients who had incomplete data or were diagnosed with severe diseases (e.g., myocardial infarction, cerebral hemorrhage, or malignancy) or infectious diseases (e.g., pneumonia or urinary infection) within 3 months were excluded. Two hundred and forty-eight patients were enrolled and 174 patients were included in this cross-sectional study. On the basis of vitamin D deficiency, the participants were grouped into control group (*n* = 69) and vitamin D deficiency group (*n* = 105). According to tertiles, modified HOMA-β was grouped three groups and 58 participants were included in each group.

### Measurement

Basic data were collected by questionnaire, which included sex, age, DM course, diabetes history, smoking, alcohol drinking, hypertension, and insulin use. Height in cm and weight in kg were measured without shoes and with light clothes, respectively, and the body mass index (BMI) was calculated using the body mass index (BMI; kg/m^2^), as follows: weight (kg) / height^2^ (m^2^). Height and weight were rounded up to the nearest 0.1 and the mean value was obtained by measuring twice and taking the average of the two measurements.

Blood samples from all participants were collected from the antecubital vein after 8 h of fasting. The same indicators for all samples were tested using one machine in one laboratory. These indicators included FBG, FCP, HbA1c, total protein, albumin, the lipid profile (total cholesterol [TC], triglyceride [TG], high-density lipoprotein cholesterol [HDL-C], low-density lipoprotein cholesterol [LDL-C]), and bone turnover biomarkers (25OHD, β-C-terminal cross-linked telopeptide of type I collagen [β-CTX], procollagen type 1 N-terminal propeptide [P1NP], and parathyroid hormone [PTH]). The modified HOMA-β was calculated as follows: 270 × FCP / (FBG − 3.5) × 0.333 (FCP in ng/mL and FBG in mmol/L).

All data were inputted by one researcher and checked by a second researcher. If errors were observed, data were checked and revised under two researchers’ supervision.

### Statistical analysis

The data were analyzed statistically using SPSS 22.0 (IBM, Inc., Armonk, NY, USA). The modified HOMA-β results were grouped into tertiles. Differences in the measurement data were compared using an analysis of variance (ANOVA) if the data had a normal distribution, and these data were expressed as the mean ± standard deviation (SD). A nonparametric test was used if the data had a skewed distribution, and these data were expressed as the median (interquartile range). A trend Chi-square test was used for counting data, and these data were expressed as the number (percentage). The association between 25OHD and modified HOMA-β was assessed using linear correlation analysis. After comparisons among different groups, cofounding factors were screened based on a *P*-value < 0.1. We used multivariate ordinal logistic regression analysis to explore the relationship of vitamin D and modified HOMA-β. Results were shown as the odds ratio (OR) and 95% confidence interval (95%CI). In Model 1, no cofounding factors were adjusted. In Model 2, the DM course, age, and BMI were adjusted. In Model 3, the DM course, age, BMI, HbA1C, TG, β-CTX, and PINP were adjusted. A *P*-value < 0.05 was considered to be statistically significant.

## Results

### Characteristics of the included population

**Fig. 1:**
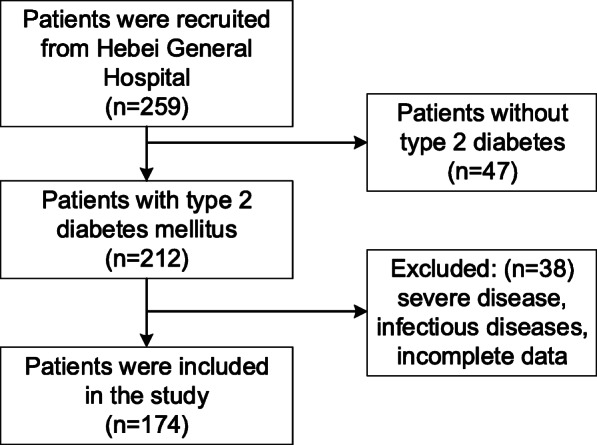
Flow diagram of participant recruitment into the study

A flow diagram for patient recruitment is shown in Fig. 1. The study population consisted 174 patients (122 men and 52 women) aged 26 to 79 with T2DM. The average HbA1c was 9.1% (7.6, 10.9). 25OHD levels ranged from 4.37 to 38.6 ng/mL. The participants’ baseline characteristics and blood indicators are shown in Table 1.

**Table 1:** Participants’ basic characteristics

	All participants
n	174
Male (n, %)	122 (68.5%)
Age (years)	53.0 ± 10.6
BMI (kg/m^2^)	25.69 ± 3.29
History of DM	64 (36.8%)
DM course (years)	
0–1	34 (14.7%)
1–10	74 (31.9%)
10–20	49 (21.1%)
≥ 20	17 (7.3%)
Hypertension	70 (40.2%)
Smoking	65 (37.4%)
Drinking	53 (30.5%)
Usage of insulin	54 (31.0%)
HbA1c (%)	9.1 (7.6, 10.9)
FBG (mmol/L)	6.49 (5.65, 7.85)
FCP (ng/mL)	1.66 (1.12, 2.47)
TC (mmol/L)	4.72 (4.06, 5.55)
TG (mmol/L)	1.43 (0.98, 2.19)
HDL-C (mmol/L)	1.05 (0.90, 1.18)
LDL-C (mmol/L)	3.09 (2.62, 3.73)
ApoA1 (mmol/L)	1.23 (1.12, 1.37)
ApoB (mmol/L)	0.80 (0.70, 0.98)
Total protein (g/L)	68.58 ± 5.80
Albumin (g/L)	41.59 ± 2.87
25OHD (ng/mL)	18.37 (13.36, 23.85)
OC (ng/mL)	12.00 (9.64, 15.61)
β-CTX (ng/mL)	0.35 (0.24, 0.52)
P1NP (ng/mL)	37.33 (29.25, 50.73)
PTH (pg/mL)	35.52 (25.00, 46.57)
Modified HOMA-β	451.30 (285.19, 633.52)

Counting data were expressed as number (percentages, %). Measurement data for normal distribution were expressed as (mean ± SD). Measurement data for skewed distribution are expressed as median (interquartile range)

*ApoA1* apolipoprotein A1, *ApoB* apolipoprotein B, *BMI* body mass index, *DM* diabetes mellitus, *FBG* fasting blood glucose, *FCP* fasting C-peptide, *HbA1c* glycated hemoglobin, *HDL-C* high-density lipoprotein cholesterol, *HOMA* homeostasis model assessment, *LDL-C* low-density lipoprotein cholesterol, *25OHD* 25-hydroxyvitamin D, *TC* total cholesterol, *TG* triglyceride, *OC* osteocalcin, *β-CTX* β-C-terminal cross-linked telopeptide of type I collagen, *P1NP* procollagen type 1 N-terminal propeptide, *PTH* parathyroid hormone

### Modified HOMA-β levels based on vitamin D deficiency

**Fig. 2:**
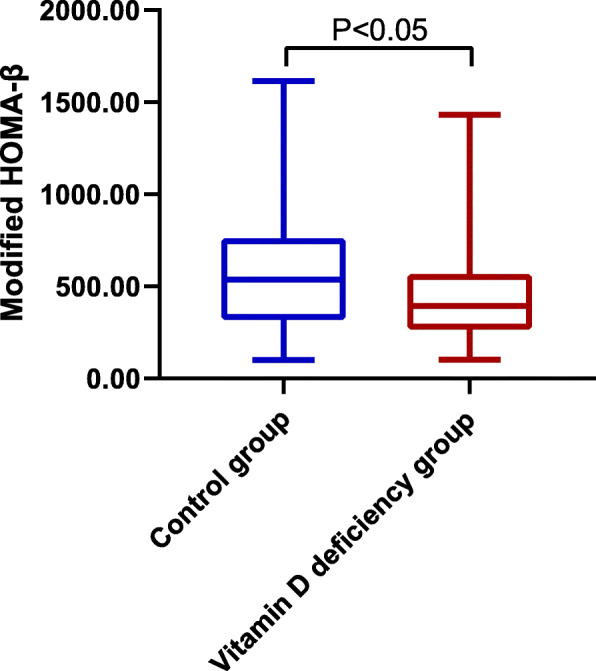
Comparison of modified HOMA-β between the control group and the vitamin D deficiency group. Note: HOMA, homeostasis model assessment

Vitamin D deficiency was defined as 25OHD < 20 ng/mL. On the basis of the presence or absence of vitamin D deficiency, the modified HOMA-β level decreased significantly in the vitamin D deficiency group compared with the control group (no vitamin D) (394.7 [259.42, 564.76] vs. 536.44 [318.65, 762.12]; *p* = 0.027; Fig. 2).

### Characteristics of the participants based on modified HOMA-β tertiles

Table 2 shows the comparisons among different modified HOMA-β groups on the basis of the tertiles. BMI was higher in T2 and T3 groups than that in T1 group (*P*-value = 0.041). There were differences in the DM course among different modified HOMA-β groups (*P*-value = 0.007). Compared with T1 group, the HbA1c level was decreased in the other two groups (*P*-value < 0.001). The FBG level decreased from the T1 group to the T3 group (*P*-value < 0.001), and FCP showed the opposite trend (*P*-value < 0.001). TG and 25OHD levels were higher in the T3 group than in the T1 and T2 groups (*P*-value = 0.003 and 0.005, respectively). We screened BMI, DM course, age, HbA1c, TG, 25OHD, β-CTX, and P1NP as confounding factors on the basis of *P* < 0.1. Because FBG and FCP were needed to calculate the modified HOMA-β, they were not seen as confounding factors.

**Table 2:** Comparisons of clinical characteristics in type 2 diabetic patients on the basis of the modified HOMA-β grouping

	T1 (*n* = 58) (99.23–338.69)	T2 (*n* = 58) (346.19–565.02)	T3 (*n* = 58) (566.74–1614.57)	*P*-value
Male (n, %)	41 (70.7%)	32 (55.2%)	49 (84.5%)	0.106
Age (years)	55.3 ± 9.1	52.1 ± 12.3	51.5 ± 9.8	0.078
BMI (kg/m^2^)	24.81 ± 3.00	26.00 ± 3.10	26.26 ± 3.61	0.041
History of DM	22 (37.9%)	21 (36.2%)	21 (36.8%)	0.848
DM course (years)				0.007
0–1	7 (12.1%)	16 (27.6%)	11 (19.0%)	
1–10	20 (34.5%)	24 (41.4%)	30 (51.7%)	
10–20	21 (36.2%)	13 (22.4%)	15 (25.9%)	
≥ 20	10 (17.2%)	5 (8.6%)	2 (3.4%)	
Hypertension	22 (37.9%)	23 (39.7%)	25 (43.1%)	0.571
Smoking	26 (44.8%)	10 (17.2%)	29 (50.0%)	0.566
Drinking	22 (37.9%)	10 (17.2%)	21 (36.2%)	0.841
Usage of insulin	19 (32.8%)	22 (37.9%)	54 (31.0%)	0.230
HbA1c (%)	10.0 (8.5, 11.3)	9. 5 (8.2, 11.0)	7.6 (6.5, 9.5)	< 0.001
FBG (mmol/L)	7.78 (6.69, 7.64)	6.35 (5.69, 7.64)	5.93 (5.36, 6.55)	< 0.001
FCP (ng/mL)	1.09 (0.78, 1.48)	1.73 (1.26, 2.21)	2.58 (1.98, 3.05)	< 0.001
TC (mmol/L)	4.76 (3.95, 5.76)	4.59 (3.95, 5.32)	4.57 (4.14, 5.44)	0.599
TG (mmol/L)	1.32 (0.91, 2.06)	1.17 (0.81, 1.92)	1.60 (1.36, 2.34)	0.003
HDL-C (mmol/L)	1.08 (0.93, 1.24)	1.07 (0.90, 1.17)	1.01 (0.87, 1.15)	0.118
LDL-C (mmol/L)	3.14 (2.57, 3.94)	3.08 (2.59, 3.66)	3.07 (2.66, 3.71)	0.871
ApoA1 (mmol/L)	1.16 (1.08, 1.36)	1.10 (0.98, 1.36)	1.20 (1.07, 1.37)	0.281
ApoB (mmol/L)	0.79 (0.68, 1.00)	0.78 (0.71, 0.95)	0.81 (0.71, 1.00)	0.794
Total protein (g/L)	68.54 ± 6.07	68.76 ± 6.04	68.23 ± 5.51	0.887
Albumin (g/L)	41.22 ± 2.90	41.62 ± 2.99	41.81 ± 2.88	0.551
25OHD (ng/mL)	16.31 (12.75, 22.14)	16.66 (12.26, 22.32)	21.05 (16.31, 25.32)	0.005
OC (ng/mL)	11.38 (9.56, 14.21)	11.70 (8.49, 15.80)	13.11 (10.57, 16.20)	0.107
β-CTX (ng/mL)	0.30 (0.22, 0.46)	0.33 (0.22, 0.53)	0.42 (0.28, 0.53)	0.057
P1NP (ng/mL)	36.60 (28.47, 45.66)	35.80 (26.79, 50.15)	41.12 (32.11, 56.50)	0.051
PTH (pg/mL)	36.18 (23.33, 46.58)	34.92 (24.21, 47.50)	35.50 (27.56, 44.53)	0.864

Counting data were expressed as number (percentages, %). Measurement data for normal distribution were expressed as (mean ± SD). Measurement data for skewed distribution are expressed as median (interquartile range)

*ApoA1* apolipoprotein A1, *ApoB* apolipoprotein B, *BMI* body mass index, *DM* diabetes mellitus, *FBG* fasting blood glucose, *FCP* fasting C-peptide, *HbA1c* glycated hemoglobin, *HDL-C* high-density lipoprotein cholesterol, *HOMA* homeostasis model assessment, *LDL-C* low-density lipoprotein cholesterol, *25OHD* 25-hydroxyvitamin D, *TC* total cholesterol, *TG* triglyceride, *OC* osteocalcin, *β-CTX* β-C-terminal cross-linked telopeptide of type I collagen, *P1NP* procollagen type 1 N-terminal propeptide, *PTH* parathyroid hormone

### Linear correlation analysis of 25OHD and modified HOMA-β

**Fig. 3:**
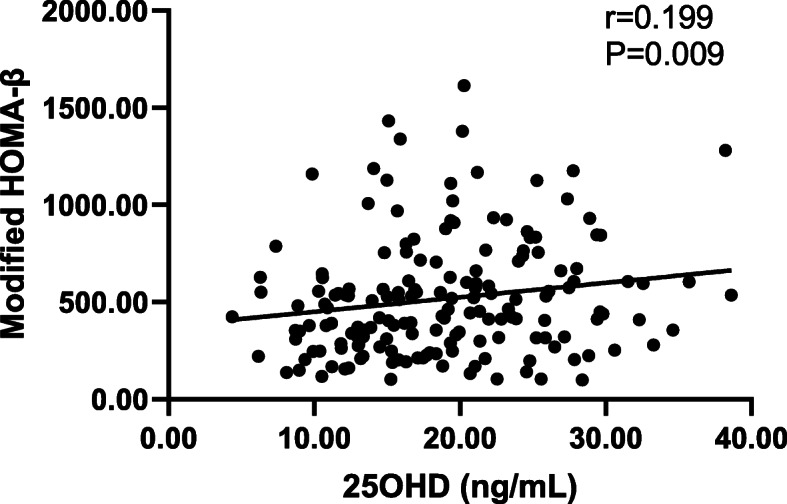
Linear correlation between 25OHD and modified HOMA-β. Note: 25OHD, 25-hydroxy vitamin D; HOMA, homeostasis model assessment.

Linear correlation analysis showed that as the 25OHD level increased, the modified HOMA-β level also increased (*r* = 0.199, *P* = 0.009; Fig. 3).

### Correlation of 25OHD and modified HOMA-β

The first ordinal logistic regression analysis was conducted where 25OHD was seen as a continuous variable (shown in Table 3). In Model 1, no confounding factors were adjusted, and this analysis showed that 25OHD increased by 1 ng/mL, and the modified HOMA-β increased by 5.7%. In Model 2, the DM course, age, and BMI were adjusted, and the modified HOMA-β increased by 6.9%. In Model 3, HbA1C, TG, β-CTX, and PINP were adjusted based on Model 2, and the age increased by 1 ng/mL and modified HOMA-β improved by 5.9%.

**Table 3:** Multivariate ordinal logistic regression analysis of relationship between 25OHD and modified HOMA-β (25OHD as continuous variable)

	Low 25OHD	Change of modified HOMA-β followed by 25OHD increases by 1 ng/mL	*P*-value
**Model 1**	Reference	1.057 (1.014, 1.101)	0.008
**Model 2**	Reference	1.069 (1.025, 1.115)	0.002
**Model 3**	Reference	1.059 (1.008, 1.112)	0.022

Model 1: No factors were adjusted

Model 2: DM course, age and BMI were adjusted

Model 3: Based on Model 2, HbA1c, TG, β-CTX and P1NP were adjusted

The data are expressed as odds ratio (95% confidence interval CI)

*BMI* body mass index, *DM* diabetes mellitus, *HbA1c* glycated hemoglobin, *HOMA* homeostasis model assessment, *25OHD* 25-hydroxyvitamin D, *TG* triglyceride, *β-CTX* β-C-terminal cross-linked telopeptide of type I collagen, *P1NP* procollagen type 1 N-terminal propeptide

A second ordinal logistic regression analysis was conducted where 25OHD was considered to be a categorical variable (no vitamin D deficiency and vitamin D deficiency) (shown in Table 4). In Model 1, no confounding factors were adjusted, and the modified HOMA-β in patients with no vitamin D deficiency increased by 2.184-fold compared with patients with vitamin D deficiency. In Model 2, when DM course, age, and BMI were adjusted, the OR was 2.520. In Model 3, HbA1c, TG, CTX, and PINP were adjusted based on Model 2, and the modified HOMA-β in patients with no vitamin D deficiency increased 2.234-fold compared with compared with those with vitamin D deficiency.

**Table 4:** Multivariate ordinal logistic regression analysis of relationship between 25OHD and modified HOMA-β (25OHD as categorical variable)

	Vitamin D deficiency	No vitamin D deficiency	*P*-value
**Model 1**	Reference	2.184 (1.236, 3.861)	0.007
**Model 2**	Reference	2.520 (1.396, 4.549)	0.002
**Model 3**	Reference	2.234 (1.153, 4.327)	0.017

Vitamin D deficiency was defined as 25OHD < 20 ng/mL

Model 1: No factors were adjusted

Model 2: DM course, age and BMI were adjusted

Model 3: Based on Model 2, HbA1c, TG, β-CTX and P1NP were adjusted

The data are expressed as odds ratio (95% confidence interval CI)

*BMI* body mass index, *DM* diabetes mellitus, *HbA1c* glycated hemoglobin, *HOMA* homeostasis model assessment, *25OHD* 25-hydroxyvitamin D, *TG* triglyceride, *β-CTX* β-C-terminal cross-linked telopeptide of type I collagen, *P1NP* procollagen type 1 N-terminal propeptide

## Discussion

In this study, we investigated the relationship between vitamin D status and modified HOMA-β. To eliminate potential confounding factors, the *P*-value was limited to 0.1. Although there was a weak relationship between 25OHD levels and modified HOMA-β, the relationship may also be affected by other factors and sample size. Previous studies have shown a correlation between these two variables [2]. We further explored the relationship between 25OHD levels and modified HOMA-β through logistic regression analysis, while considering potential confounding factors. In the analysis process, we considered 25OHD to be a continuous and categorical variable, and we conducted a multivariate ordinal logistic regression analysis twice to obtain better and more plausible results. Whether or not confounding factors were adjusted, the results showed that 25OHD was positively related to the modified HOMA-β, meaning that, with an increase in 25OHD, the modified HOMA-β function improved. In addition, the relationship between 25OHD and fat mass is controversial. Some studies have shown that 25OHD levels and fat mass are negatively related [9], whereas other studies have not shown this relationship. Therefore, fat mass may be a confounder for the final results [10]. However, we did not measure the patients’ fat mass in our study.

Beyond the effect of vitamin D on calcium and bone metabolism, the role of vitamin D on IR, glucose metabolism, and T2DM has received widespread attention. Vitamin D can regulate insulin secretion and mediate pancreatic β cell survival, which was shown in previous studies [11, 12].

The mechanisms of vitamin D that regulate islet function may be involved in several aspects. First, the vitamin D receptor (VDR) was expressed in pancreatic β cells, and vitamin D can exert its effect by directly binding to the VDR [13]. Mice lacking VDR showed impairment in insulin secretion after glucose loading [14]. Vitamin D can also promote insulin secretion because it can identify the insulin gene promotor’s vitamin D-responsive elements (VDRE). The VDRE regulates insulin gene transcription and is involved in intracellular junctions and cellular growth in pancreatic β cells [15]. Second, 25(OH)D-1α-hydroxylase (CYP27B1) is also expressed in pancreatic β cells, which is an enzyme that mediates 25OHD to transform it into the active 1,25(OH)_2_D_3_. Third, intracellular calcium (Ca^2+^) levels are essential for insulin action. Vitamin D is involved in regulating Ca^2+^ flux in pancreatic cells [16]. Fourth, vitamin D can act on pancreatic β cells and on insulin-sensitive tissues, such as white adipose tissue, muscle, and liver to regulate insulin sensitivity [11]. For example, 1,25(OH)_2_D_3_ improves glucose metabolism via the SIRT1/IRS1/GLUT4 signaling cascade in C2C12 myotubes [17]. In addition, vitamin D is indirectly related to oxidative stress [18], sub-inflammation [19], and epigenetics [20].

The hyperinsulinemic–euglycemic clamp is the gold standard that is currently used to quantitatively evaluate insulin secretion and IR. Its accuracy and stability are widely recognized. However, because of the invasiveness of surgery and technical difficulties, its use has been limited. In clinical studies, HOMA-IR and HOMA-β are an evaluation method that uses fasting glucose and insulin levels. Patients with T2DM, especially patients with poor islet function, usually inject insulin subcutaneously to have good glycemic control. Therefore, the modified HOMA-β is need for these patients.

The current study has some limitations. First, for cross-sectional studies, our sample size was small. The results are more convincing when there is a large sample size in a cross-sectional observational study. Second, exposure to sunshine, physical exercise, vitamin D, calcium intake, or drugs that treat osteoporosis may be confounding factors, but we did not take these factors into account. Third, because of this study type, we could not obtain a causal relationship between vitamin D and islet function. Fourth, patients with or without insulin therapy were all included in this study. It is more appropriate to recruit patients without insulin therapy to compare the traditional HOMA-β and modified HOMA-β.

## Conclusions

In conclusion, taking all the potential confounding factors into account, the vitamin D status and modified HOMA-β showed a positive relationship. Thus, an increase in or supplementation of vitamin D may be related to improved modified HOMA-β function and delayed pancreatic β cell functional decline.

## Supplementary Information

**Additional file 1.** Questionnaire.

## Data Availability

The original data can be obtained by email request (hangzhao4006@163.com).

## Abbreviations

ANOVA: Analysis of variance
ApoA1: Apolipoprotein A1
ApoB: Apolipoprotein B
BMI: Body mass index
CI: Confidence interval
CYP27B1: 25(OH)D-1α-hydroxylase
DM: Diabetes mellitus
FBG: Fasting blood glucose
FCP: Fasting C-peptide
HbA1c: Glycated hemoglobin
HDL-C: High-density lipoprotein cholesterol
HOMA: Homeostasis model assessment
LDL-C: Low-density lipoprotein cholesterol
25OHD: 25-hydroxyvitamin D
SD: Standard deviation
TC: Total cholesterol
TG: Triglyceride
OC: Osteocalcin
OR: Odds ratio
β-CTX: β-C-terminal cross-linked telopeptide of type I collagen
P1NP: Procollagen type 1 N-terminal propeptide
PTH: Parathyroid hormone
VDR: Vitamin D receptor
VDRE: Vitamin D-responsive elements
