# Supplementary material for: The relationship between vitamin D status and islet function in patients with type 2 diabetes mellitus
Source: BMC Endocr Disord. 2021 Oct 18;21:203. doi: 10.1186/s12902-021-00862-y (PMC8522231; doi:10.1186/s12902-021-00862-y)
Supplement: Supplementary file 1 — Additional file 1. Questionnaire. [file 12902_2021_862_MOESM1_ESM.pdf]

Questionnaire

Basic Characteristics

- **First Name**________ **Last Name**_________
- **Hospital Admission Number**________
- **Hospital Admission Date**________
- **Sex**: M/F **Age**_____(year)
- **Diabetes Course**_____(y / m / w) **Diabetes History**: Y/N
- **Smoking**: Y/N **Alcohol drinking**: Y/N
- **Hypertension**: Y/N **Insulin Use**: Y/N
- **Height**______cm **Weight**______kg

Blood indicators

FBG_______mmol/L **FCP**_______ng/mL

Total Protein_______g/L **Albumin**_______g/L

Lipid:

TC_______mmol/L **TG**_______mmol/L

HDL-C_______mmol/L **LDL-C**_______mmol/L

ApoA1_______mmol/L **ApoB**_______mmol/L

Bone turnover biomarkers:

25OHD_______ng/mL **OC**_______ng/mL

β-CTX_______ng/mL **P1NP**_______ng/mL

PTH_______pg/mL

Researcher Signature_______________

Date_______________
